# Exposure to wartime sexual violence in Bosnia and Herzegovina: nationally representative prevalence 30 years after the 1992–1995 war

**DOI:** 10.1186/s13031-025-00741-6

**Published:** 2025-12-23

**Authors:** Max Schaub, Alina Greiner-Filsinger, Ajla Henic, Lennart Kasserra

**Affiliations:** 1 University of Hamburg, Allende-Platz 1, 20146 Hamburg, Germany; 2 WZB Berlin Social Science Center, Berlin, Germany; 3 University of Mannheim, Mannheim, Germany; 4 University of Hamburg, Hamburg, Germany; 5 University of Konstanz, Konstanz, Germany

## Abstract

**Background:**

Although Bosnia and Herzegovina is one of the most studied cases of wartime sexual violence, reliable population-based data on such violence remain lacking. This study provides nationally representative prevalence estimates of direct, family-level, and community-level exposure to sexual violence nearly 30 years after the 1992–1995 war, and reports descriptive associations with selected psychosocial indicators.

**Methods:**

We conducted a face-to-face household survey of 2,059 adults in 2024 using stratified, multi-stage sampling. Personal, family, and community exposure to wartime sexual violence were measured as part of a module recording victimization experiences. To assess potential underreporting, we included list experiments as an indirect measure.

**Results:**

Personal exposure to wartime sexual violence was reported by $1.6\%$ of respondents, while $6.0\%$ reported family exposure and $11.3\%$ indicated that someone in their community had been victimized. Family exposure varied sharply by ethnicity, with $10.2\%$ among Bosniak (Bosnian Muslim), $1.5\%$ among Croat, and $1.1\%$ among Serb respondents. List experiment results yielded comparable estimates: $2.4\%$ for personal exposure and $9.6\%$ for family exposure. Respondents reporting exposure exhibited lower well-being, poorer self-rated health, more frequent sleep disturbances, and higher levels of domestic violence.

**Conclusions:**

These findings provide the first robust, nationally representative estimates of wartime sexual violence exposure in Bosnia and Herzegovina. They underscore the enduring social and health consequences of wartime sexual violence and highlight the need for sustained mental health and social support interventions to address its intergenerational legacy.

**Supplementary Information:**

The online version contains supplementary material available at 10.1186/s13031-025-00741-6.

## Introduction

Reliable population-based data on wartime sexual violence remain scarce [1, 2]. Bosnia and Herzegovina (BiH) is no exception. The 1992–1995 war, which followed the breakup of Yugoslavia, exposed civilians to severe and varied forms of violence, including systematic campaigns of ethnic cleansing and mass atrocities, and caused at least 100,000 war-related deaths [3–7]. Sexual violence was perpetrated across the conflict against both women and men, often in detention and rape camps, serving as an instrument of ethnic persecution and patriarchal domination [8–19].

Yet, despite extensive documentation by journalists, humanitarian organizations, and courts of law [15, 20], no nationally representative estimates of the prevalence of wartime sexual violence in BiH are available. Existing figures, often cited in public discourse, stem from early wartime estimates or small, non-representative samples of survivors [21–27] and therefore cannot be used to infer the broader population burden. The commonly referenced estimates of 20,000 to 50,000 documented female victims and around 3,000 male victims provide only a rough sense of the scale of the violence and rest on case reports rather than representative data [18, 19, 22, 28, 29]. The continued absence of systematic evidence stands in contrast to the importance of the Bosnian case, which reshaped the public debate on wartime sexual violence [16, 30, 31], and to the need for understanding how such violence reverberates across families and communities over time [8, 32].

This study provides the first nationally representative estimates of exposure to wartime sexual violence in Bosnia and Herzegovina, measured nearly 30 years after the end of the conflict. We report prevalence rates of personal, family-level, and community-level exposure to wartime sexual violence, and assess how these experiences continue to correlate with indicators of well-being, health, and domestic violence. The findings show that exposure to wartime sexual violence remains a widespread and consequential aspect of post-conflict life in Bosnia and Herzegovina, with lasting associations across multiple domains of health and psychosocial well-being [32–34]. By providing population-level estimates of the prevalence and distribution of wartime sexual violence nearly three decades after the conflict and in a setting that has come to define global understandings of such violence, this study offers a rare, long-term view of its enduring presence within the population.

## Data and methods

### Data

Data collection was carried out face-to-face during September to December 2024 by a reputable survey company using computer-assisted personal interviewing (CAPI). Respondent recruitment followed a multi-stage random sampling procedure to select enumerator starting points, followed by a random walk. The primary sampling units (PSUs) were Bosnia’s 6,141 *naselja* (settlements), the smallest administrative unit used in the country. We drew a random population-weighted sample of 162 PSUs using population statistics provided by the Agency for Statistics of Bosnia and Herzegovina (ASBH). For the original draw, we stratified the population into strata with higher and lower risk of sexual victimization, and oversampled the former. Our analyses therefore include design weights that correct this oversampling. By scaling the weights to the target population size, we also reduce potential bias arising from differential non-response across strata and settlement sizes. Within each settlement we determined enumerator starting points. For this purpose, settlements were overlaid with a 0.006 degree raster grid. We then drew raster cells by means of another population-weighted random draw, with grid cell populations estimated using the Global Human Settlement Layer (GHSL), produced by the European Commission’s Joint Research Centre [35]. Enumerator starting points were roadside locations within the selected grid cells. Individual respondent recruitment then followed a random walk procedure with enumerators contacting every third household. One interview per household was conducted with a household member over 18 years with the most recent birthday. Interviews lasted 28 minutes on average. In total, 5,288 recruitment attempts were made for a final sample of 2,059 completed interviews and a response rate of 38.9%, similar to comparable social surveys conducted in Bosnia and Herzegovina [36, 37]. Additional information about the survey, in line with the STROBE reporting guidelines [38], can be found in Section B in the Supporting Information (SI).

### Ethics

Given the sensitive nature of the survey, we took several measures to mitigate potential harm to respondents. Before the start of the interview, respondents were briefed about the nature of the questionnaire and could refuse participation upfront; they could also choose to skip individual sensitive items or to terminate the interview at any point. To allow respondents privacy when responding to the sensitive items, enumerators handed over the tablet, enabling respondents to answer questions on their own, in line with recommendations for interviewing victims of WTSV [31]. The survey was programmed so that once respondents privately entered their answers to the sensitive items, the enumerator could not return to or view these questions, thereby ensuring privacy and minimizing social pressure. Enumerators received training in trauma-informed interviewing and were instructed to monitor verbal and non-verbal cues of distress, to pause or discontinue the interview if needed, and to report any concerns during daily debriefings with field supervisors. A formal referral protocol to a Sarajevo-based psychologist specializing in wartime trauma was in place throughout fieldwork, and all respondents received a written debriefing sheet with our contact details, the psychologist’s number, and relevant local helplines, ensuring access to support independent of the enumerator. No respondent required activation of the referral mechanism. Despite these safeguards, the non-activation of the referral protocol cannot be interpreted as definitive evidence that no discomfort occurred, given the stigmatized nature of the subject matter. Further methodological work on detecting and responding to participant distress in such contexts would be valuable. All procedures were coordinated with and approved by the WZB Berlin Social Science Center’s Research Ethics Review Board (Decision Nr. 2024/03/240).

### Measurement

Our outcomes of interest are the prevalence of individual (where applicable), family member, and community exposure to wartime sexual violence. Exposure to sexual violence was measured as part of a ten-item victimization module asked midway through the survey. Respondents were asked: “During the period of the war in the 1990 s, which of the following, if any, happened to you, to members of your family or community? I will hand over the device to you to give you privacy. If a particular event happened to you or to someone in your family or community—or you suspect that it happened—make a cross; otherwise leave empty.” They were then presented with a list of different types of wartime victimization ranging from “Had house, business, or fields destroyed” and “Forced out of home” to “Shot, shelled, bombed” and, of interest here, “Subjected to sexual violence.” The module was pre-tested to be mutually exclusive and exhaustive, and the full distribution of responses is reproduced in Table S2 in the SI. For each type of wartime victimization, including exposure to wartime sexual violence, respondents could indicate whether it happened to them, to someone in their family, or to someone in their community (or refuse to answer). Direct questions about personal wartime experiences were asked only of respondents who were at least 12 years old at the beginning of the 1992–1995 war (i.e., those aged 44 or older in 2024), ensuring that solely individuals who could plausibly have been directly exposed were included in the denominator for individual-level prevalence estimates. This is reflected in the lower sample size of n=968 for these items (see Table S2 in the SI). Younger respondents were asked about family- and community-level exposure only. In other parts of the survey, we recorded respondents’ sex and ethnicity, allowing us to stratify responses by these dimensions.

### List experiment

A major methodological concern with recording highly sensitive information such as exposure to wartime sexual violence by means of direct questions is potential non-response bias or respondents not answering truthfully. While the privacy-enhancing questioning methods mitigate this concern, we additionally implemented two list experiments, also known as item count technique (ICT) [39–42]. For the first list experiment, a randomly selected half of respondents (assigned by the CAPI device), the treatment group, were asked “Can you tell me how many of these experiences happened to you during the war in the 1990s?” and given a four-item list that read “I had to leave my home”, “I was part of a prisoner’s exchange”, “I witnessed someone being killed”, and “I was subjected to sexual violence”. The control group was given the same list minus the item “I was subjected to sexual violence”. Respondents were then asked to report “how many of these experiences happened to you during the war in the 1990s?” and explicitly informed that they would not need to say which things happened to them, just how many. The difference of the average number of items mentioned by the intervention group and the control group then gives an estimate of the prevalence of exposure to wartime sexual violence in our sample. For the second list experiment, an analogous question was asked focused on family victimization.

**Table 1:** **Summary statistics sample demographics**

	Mean	SD	Min	Max	Count
Female	0.49	0.50	0.00	1.00	2,059
Age	45.91	16.49	18.00	85.00	2,059
Bosniak	0.50	0.50	0.00	1.00	2,059
Serb	0.35	0.48	0.00	1.00	2,059
Croat	0.15	0.36	0.00	1.00	2,059

Weighted summary statistics for core demographic variables. Full descriptive results, including all outcome variables, are provided in Table S2 in the Supplementary Information (SI)

### Analysis strategy

For the self-reported prevalence rates, we report weighted averages and corresponding 95% confidence intervals (CIs). Confidence intervals are constructed on the logit (log-odds) scale and back-transformed to the probability scale, a standard approach for estimating prevalence rates (proportions), which are naturally restricted to the 0–1 interval [43–45]. Prevalence rates are displayed graphically, with numeric values reported in the text. The analysis was conducted in Stata 19 using the software’s “svy” command to account for the complex sampling design and to estimate uncertainty.

For the list experiment, we estimate ordinary least squares (OLS) regressions of the number of items mentioned on a binary indicator for assignment to the treatment or control group, including primary sampling unit (PSU) fixed effects to account for spatially driven heterogeneity. The regression coefficient on the treatment indicator represents the experimental estimate of prevalence. Confidence intervals for the list-experimental estimates are derived analytically, while differences between the direct and list-experimental estimates are assessed using nonparametric bootstrapping [46].

## Results

### Summary statistics

Table 1 presents weighted descriptive statistics for key demographic characteristics of the sample. Approximately half of our sample (49%) was female, with an average age of 46 years. This compares to a projected average age of the 18+ years population of 48 years for the year 2020 [47]. Ethnic composition in the sample—50% Bosniak, 35% Serb, and 15% Croat—closely matches the 2013 census proportions of 50%, 31%, and 15% [48], indicating that the sampling strategy successfully captured population composition.

**Fig. 1:**
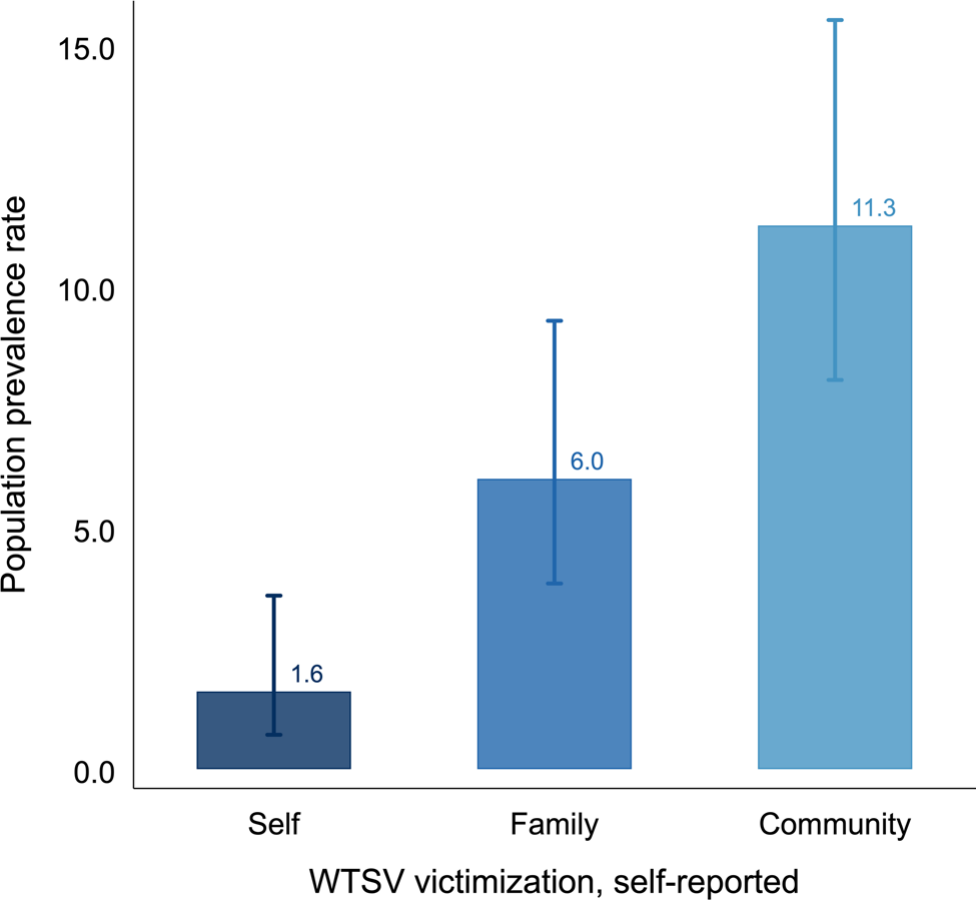
**Population prevalence for exposure to wartime sexual violence**. Prevalence of individual-, family-, and community-level exposure to wartime sexual violence (WTSV) in Bosnia and Herzegovina, self-reported. Bars show weighted population prevalence rates; lines indicate logit-based 95% confidence intervals

### Prevalence rates

Figure 1 presents the core outcome of this study: the prevalence of exposure to wartime sexual violence (WTSV) nearly thirty years after the end of the war in Bosnia and Herzegovina. Overall, $1.61\%$ of respondents (95% CI: 0.71 to 3.60) reported that they were personally subjected to sexual violence during the war; $6.02\%$ (95% CI: 3.85 to 9.30) reported that a family member had experienced WTSV; and $11.27\%$ (95% CI: 8.07 to 15.53) indicated that WTSV had taken place within their communities. These estimates, derived from a population-representative survey, thus indicate that sexual violence affected a non-negligible share of the population, with more than one in ten respondents reporting that members of their community were victims of WTSV.

### Prevalence by subgroup

**Fig. 2:**
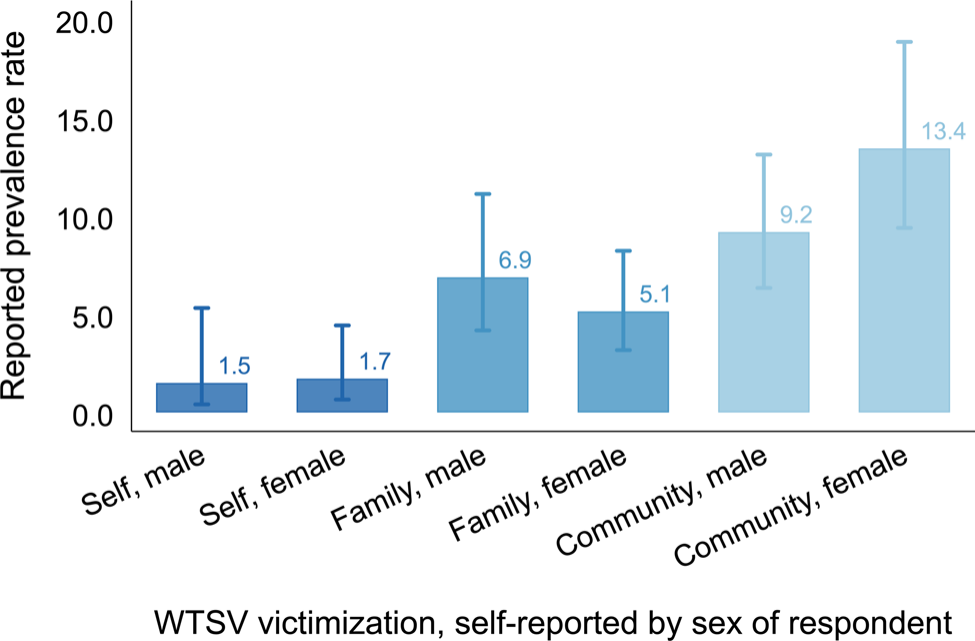
**Prevalence of WTSV by sex**. Personal, family, and community exposure to wartime sexual violence (WTSV) by sex of respondent. Bars show weighted mean prevalence rates; lines indicate logit-based 95% confidence intervals

Figure 2 disaggregates these reported prevalence rates by the sex of the respondent. Female respondents reported slightly higher personal exposure to WTSV ($1.72\%$, 95% CI: 0.66 to 4.43) than male respondents ($1.50\%$, 95% CI: 0.41 to 5.32), but this difference is not statistically significant ($\Delta = 0.14$, $p = 0.86$). In contrast, for family-level victimization, prevalence among female respondents ($5.14\%$, 95% CI: 3.17 to 8.22) was somewhat lower than among male respondents ($6.87\%$, 95% CI: 4.17 to 11.12)—a difference that is again not statistically significant ($\Delta = 1.73$, $p = 0.16$).

The only significant difference appears for community-level exposure: $13.43\%$ of female respondents (95% CI: 9.39 to 18.86) reported that members of their community were subjected to WTSV, compared to $9.18\%$ (95% CI: 6.33 to 13.12) among male respondents ($\Delta = 4.26$, $p = 0.007$). Overall, these results thus suggest broadly similar reporting patterns across sexes, with a modestly higher propensity among women to recognize WTSV at the community level.

**Fig. 3:**
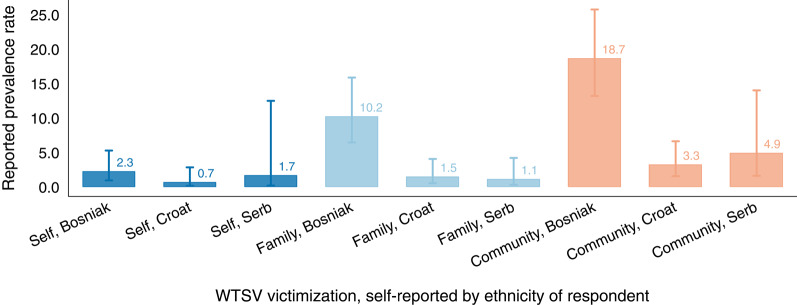
**Prevalence of WTSV by ethnicity**. Individual-, family-, and community-level exposure to wartime sexual violence (WTSV) by ethnicity of respondent. Ethnicity category “other” (16 respondents, 0.78% of the sample) omitted for ease of presentation. Bars show weighted mean prevalence rates; lines indicate logit-based 95% confidence intervals

Stark differences emerge when stratifying prevalence rates by the ethnicity of the respondent (Figure 3). Previous qualitative and documentary evidence indicates that Bosniaks were disproportionately affected by wartime sexual violence [8, 28, 49, 50], and our representative prevalence estimates closely reflect this pattern. An estimated $2.27\%$ of Bosniak respondents (95% CI: 0.96 to 5.28) reported having personally been subjected to WTSV, compared to $1.70\%$ among Croat (95% CI: 0.21 to 12.49) and $0.70\%$ among Serb respondents (95% CI: 0.17 to 2.85). Even more strikingly, $10.24\%$ of Bosniaks (95% CI: 6.46 to 15.86) reported that a close family member had been victimized—seven to nine times higher than among Croats ($1.15\%$, 95% CI: 0.30 to 4.21) or Serbs ($1.50\%$, 95% CI: 0.55 to 4.07).

The same pattern holds for community-level exposure, reported by $18.66\%$ of Bosniaks (95% CI: 13.19 to 25.73), compared with $4.94\%$ of Croat (95% CI: 1.63 to 14.01) and $3.26\%$ of Serb respondents (95% CI: 1.57 to 6.63). Differences in reported WTSV rates between Bosniak and other ethnicities are not statistically significant for personal victimization ($p> 0.1$) but are highly significant for family and community exposure ($p < 0.001$). Taken together, these results underline that Bosnia and Herzegovina’s Bosniak population experienced markedly higher rates of wartime sexual violence than other ethnic groups.

### List experiment

**Fig. 4:**
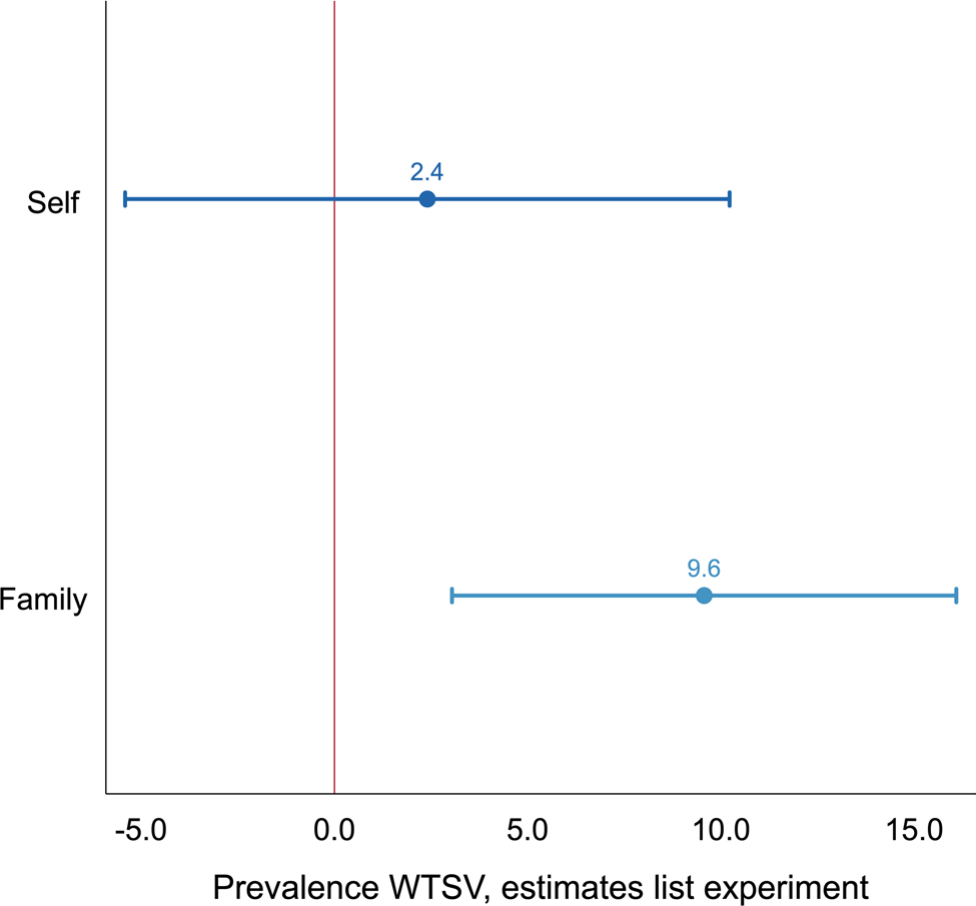
**Prevalence estimates from the list experiments**. List experiment (indirect) estimates of individual- and family-level exposure to wartime sexual violence (WTSV). Points represent mean prevalence estimates from OLS models; horizontal lines show analytical (Wald) 95% confidence intervals

To assess whether the sensitive nature of the questions may have led to underreporting, Figure 4 compares prevalence estimates derived from the list experiments with those based on direct self-reports. To minimize respondent burden, the list experiment was restricted to personal and family member WTSV victimization and did not include community member exposure.

Comparing the average number of items mentioned in the treatment and control groups yields an estimated prevalence of 2.40 percentage points (95% CI: $-5.42$ to 10.22, $p = 0.55$, not significant) for personal WTSV exposure and 9.57 percentage points (95% CI: 3.04 to 16.09, $p = 0.004$) for family WTSV victimization. These values are somewhat higher than those derived from the direct self-report questions (Figure 1), but of broadly similar magnitude. The slightly higher point estimates may reflect underreporting in direct questions, or may simply be due to sampling variability. Formal bootstrap tests reveal no statistically significant differences between the direct and list-experimental estimates for either personal WTSV exposure ($\Delta = 0.79$, $p = 0.84$) or family exposure ($\Delta = 3.55$, $p = 0.29$). The list-experimental results therefore provide independent confirmation of the reliability of the self-reported prevalence rates.

### Correlation with well-being and domestic violence

**Fig. 5:**
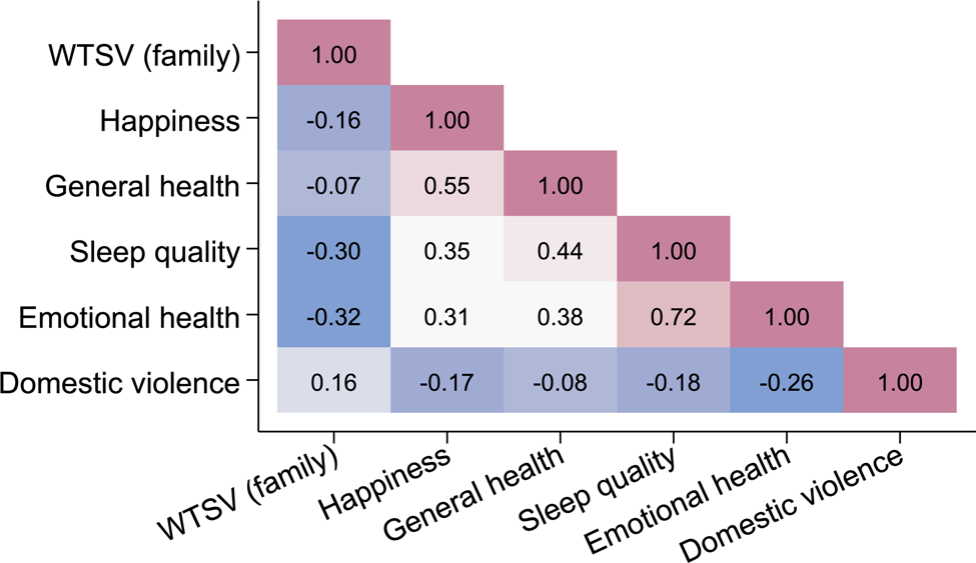
**Correlation of family exposure to wartime sexual violence with measures of well-being and domestic violence**. Pairwise correlations between family exposure to wartime sexual violence (WTSV) and indicators of well-being and domestic violence. All correlations statistically significant at $p<0.001$

As a final step in our analysis, we investigate correlates of exposure to WTSV across indicators of well-being, health, and domestic violence—dimensions that prior literature has shown to be negatively affected by such violence [24, 34, 51–58]. That literature has tended to focus on short-term outcomes, whereas our investigation contributes estimates nearly 30 years after the end of the conflict. To keep the analysis focused, we examine correlates of family-level WTSV victimization, for which complete data were available for all respondents. Figure 5 presents these main results; Figures S1 and S2 in the SI show analogous and substantively similar patterns for personal and community-level victimization.

Exposure to family WTSV is systematically associated with lower reported happiness ($\rho =-0.16$, 95% CI: $-0.21$ to $-0.12$) and poorer self-assessed health ($\rho =-0.07$, 95% CI: $-0.12$ to $-0.03$). The strongest correlations appear for sleep quality ($\rho =-0.30$, 95% CI: $-0.34$ to $-0.26$)—an indicator often associated with trauma [59, 60]—and for emotional health ($\rho =-0.32$, 95% CI: $-0.36$ to $-0.28$). Family WTSV also correlates positively with higher reported levels of domestic violence ($\rho =0.16$, 95% CI: 0.12 to 0.20). While these correlations are moderate in magnitude, they consistently point to poorer well-being among respondents reporting family-level WTSV exposure; all are statistically significant at $p<0.001$. Even 30 years after the end of the war in Bosnia and Herzegovina, WTSV exposure remains associated with a broad range of negative psychosocial outcomes.

## Discussion

This study provides the first nationally representative evidence on exposure to wartime sexual violence (WTSV) in Bosnia and Herzegovina nearly three decades after the end of the 1992–1995 war. In our 2024 survey of more than two thousand respondents, $1.6\%$ reported having personally experienced sexual violence during the war, $6.0\%$ reported that a close family member had been victimized, and $11.3\%$ indicated that such violence had occurred within their communities. Expressed in absolute numbers, these proportions correspond to approximately 27,100 individuals (95% CI: 4,986 to 49,291) who personally survived wartime sexual violence, 208,400 (95% CI: 117,385 to 299,458) who reported family-level victimization, and 390,300 (95% CI: 265,537 to 515,007) who were exposed to community-level WTSV. While these estimates are subject to uncertainty, they fall broadly within the often-cited range of 20,000 to 50,000 direct victims [18, 21, 22, 28]. Unlike those extrapolations based on case documentation, the present analysis draws on systematic, population-representative data, thereby strengthening the empirical foundation for understanding the scale of wartime sexual violence in Bosnia and Herzegovina.

Our estimates are consistent across male and female respondents, but also show that the burden of victimization is unevenly distributed: reports of family and community exposure are markedly higher among Bosniak respondents than among Croat or Serb respondents, mirroring earlier documentary evidence on patterns of violence [14, 16, 28, 49, 61]. Moreover, exposure to WTSV remains systematically associated with poorer well-being, reduced self-rated health, lower emotional stability, and higher reported domestic violence, underscoring the continuing psychosocial consequences for affected families and communities [34, 62].

The estimates presented here are necessarily subject to uncertainty and potential biases. First, non-response may lead to conservative estimates if individuals with direct or family-level exposure were less likely to participate. While this possibility cannot be fully ruled out, enumerators recorded very few refusals to the victimization module itself, suggesting that selection among respondents was limited. Second, although our stratified design intentionally oversampled high-risk localities, the use of design weights restores representativeness at the population level. Third, self-reported measures of sexual violence may suffer from underreporting, particularly in a social context where stigma persists [63]. That said, the use of indirect list experiments yielded results broadly consistent with those from direct survey questions, suggesting that underreporting was limited. The distance in time from the war may also have facilitated more candid responses by reducing fear and shame. A final source of potential bias is survivorship bias: victims of WTSV who were killed cannot report [18]. This is why family- and community-level exposure measures are essential, as they provide a more complete account of victimization, encompassing cases where direct victims survived and where they did not. Taken together, while the estimates appear robust, the potential sources of bias identified above would tend to depress rather than inflate observed prevalence. The reported figures should therefore be interpreted as conservative lower bounds.

The findings corroborate earlier qualitative and documentary evidence that sexual violence during the Bosnian war was both widespread and targeted, while providing, for the first time, systematic evidence on the scale and distribution of victimization in the population. By using non-intrusive measures and combining direct questions with list-experimental estimates to produce population-level estimates, our study directly addresses challenges highlighted in recent reviews of wartime sexual violence, including the scarcity of reliable prevalence data and the need for improved methodologies capable of capturing underreporting and hidden victimization [22, 31, 64]. Our results further speak to emerging work on the broader and longer-term consequences of wartime sexual violence for survivors and their families [65, 66]. Echoing this literature, we demonstrate that the effects of wartime sexual violence extend beyond direct survivors, affecting families and communities more broadly. Correlations with reduced well-being and elevated domestic violence underscore the enduring reach of wartime sexual violence, which continues to shape households nearly thirty years after the conflict.

These long-term consequences likely operate through multiple channels, including individual psychological sequelae and socially transmitted forms of trauma [34, 52, 54, 55, 59–62]. Addressing such long-term, intergenerational consequences requires sustained attention beyond the immediate post-conflict period. Evidence from other settings indicates that effective responses depend on enabling survivors to safely disclose their experiences and access help, building community awareness of available support, and mobilizing local actors to collectively confront and sanction sexual violence [67, 68].

Reliable data on wartime sexual violence remain scarce, even though such violence is widespread across conflicts, with systematic reviews estimating its prevalence among women and girls at roughly 11% globally [2]. Our population-level estimates fall within this global range, suggesting that Bosnia and Herzegovina’s prevalence levels are broadly comparable to those observed in other conflict settings. Similar long-term legacies to those documented in BiH are therefore likely present in other societies affected by wartime sexual violence. Systematic, population-based research across such contexts remains essential for understanding the enduring social and health burden of wartime sexual violence.

Despite the constraints of retrospective self-reporting, the results presented here offer one of the few population-level estimates of a form of violence seldom captured in representative data. Wartime sexual violence in Bosnia and Herzegovina was not only a defining feature of the war itself but continues to shape the health and well-being of individuals and families decades later.

## Supplementary Information

Supplementary file 1.

## Data Availability

De-identified survey data and replication code are available on OSF at https://osf.io/7zksg. Sensitive identifiers have been removed to protect respondent confidentiality.

## References

[1] Bendavid E, et al. The effects of armed conflict on the health of women and children. Lancet. 2021;397(10273):522–32. 10.1016/S0140-6736(21)00131-8. 33503456 10.1016/S0140-6736(21)00131-8 PMC7612212

[2] Murphy M, et al. Experience of intimate partner violence and non-partner sexual violence in conflict-affected settings: a systematic review and meta-analysis. Trauma Violence Abuse. 2024. 10.1177/15248380241305355. 39717938 10.1177/15248380241305355 PMC12569134

[3] Tabeau E, Bijak J. War-related deaths in the 1992–1995 armed conflicts in Bosnia and Herzegovina: a critique of previous estimates and recent results. Eur J Popul. 2005;21(2):187–215.

[4] Kalyvas, Stathis N, Sambanis N Bosnia’s CivilWar: Origins and Violence Dynamics. Understanding Civil War Evidence and Analysis. Volume 2, Europe, Central Asia, and Other Regions. Ed. by Paul Collier and Nicholas Sambanis. Washington, DC: World Bank, 2005:191–230.

[5] Weidmann NB. Violence from above or from below? The role of ethnicity in Bosnia’s civil war. J Polit. 2011;73(4):1178–90.

[6] Tokaèa M. The Bosnian Book of the Dead: Human Losses in Bosnia and Herzegovina 1991–1995. Sarajevo: Istraživaèko Dokumentacioni Centar Sarajevo; 2012.

[7] Vukušiæ I Serbian Paramilitaries and the Breakup of Yugoslavia: State Connections and Patterns of Violence. London: Routledge. 2022; 230.

[8] Skjelsbak I. Victim and survivor: narrated social identities of women who experienced rape during the war in Bosnia-Herzegovina. Fem Psychol. 2006;16(4):373–403. 10.1177/0959353506068746.

[9] Oosterhoff P, Zwanikken P, Ketting E. Sexual Torture of Men in Croatia and Other Conflict Situations: An Open Secret. Reproductive Health Matters. 2004;12(23):68–77 PMID: 15242212. 15242212 10.1016/s0968-8080(04)23115-9

[10] Becirbašiæ B. ’We Must Keep Talking about Rape in War’: Interview. Kvinna till Kvinna Foundation. 2022. (visited on 11/04/2025). https://kvinnatillkvinna.org/2022/06/19/we-mustkeep-talking-about-rape-in-war/

[11] Skjelsbak I. The Elephant in the Room: An Overview of How Sexual Violence Came to Be Seen as a Weapon of War. Oslo: PRIO; 2010.

[12] Milenkovska S. Hague Tribunal’s Blind Spots Marred Wartime Sexual Violence Cases. Balkan Investigative Reporting Network. 2023. (visited on 11/04/2025). https://balkaninsight.com/2023/01/24/hague-tribunals-blind-spots-marred-wartime-sexual-violence-cases/

[13] Šiljak ZS. Victim or Survivor? Choosing Identity after Wartime Sexual Violence. Healing and Peacebuilding after War Transforming Trauma in Bosnia and Herzegovina. Ed. by Marie E. Berry, Julianne Funk, and Nancy Good. Routledge Studies in Peace and Conflict Resolution. London: Taylor & Francis Ltd, 2023:121–133.

[14] Skjelsbak I. The Political Psychology of War Rape. 0th ed. Routledge, 2012. 10.4324/9780203695616.

[15] Clark Janine Natalya. Rape, Sexual Violence and Transitional Justice Challenges: Lessons from Bosnia Herzegovina. 1st ed. Routledge; 2017.

[16] Boesten J. Revisiting methodologies and approaches in researching sexual violence in conflict. Social Politics: International Studies in Gender, State & Society. 2018;25(4):457–68. 10.1093/sp/jxy035.

[17] Woodward Susan L. Balkan Tragedy: Chaos and Dissolution after the Cold War. Brookings Institution Press; 1995.

[18] Elisabeth Jean Wood. Variation in Sexual Violence during War. Politics & Society. 2006;34(3):307–42. 10.1177/0032329206290426.

[19] Touquet H. Silent or inaudible? Male survivor stories in Bosnia-Herzegovina. Social Politics International Studies in Gender State & Society. 2022;29(2):706–28.

[20] OSCE. War Crimes Case Processing in Bosnia and Herzegovina (2004 – 2023). OSCE Mission to Bosnia and Herzegovina, 2025.

[21] United Nations Security Council. Report of the Commission of Experts Established Pursuant to United Nations Security Council Resolution 780 (1992). S/1994/674. Accessed 13 November 2025. New York: United Nations. 1994.

[22] Palermo T, Peterman A. Undercounting, Overcounting and the Longevity of Flawed Estimates: Statistics on Sexual Violence in Conflict. Bulletin of the World Health Organization. 2011;89(12):924–5. 10.2471/BLT.11.089888. 22271951 10.2471/BLT.11.089888 PMC3260900

[23] Zenica M, Mondiale M. Research on the Long-Term Consequences of War Rape and Coping Strategies of Survivors in Bosnia and Herzegovina - We Are Still Alive. We Have Been Harmed but We Are Brave and Strong. Zenica: Medica Zenica; 2014. p. 171.

[24] Lonèar M, et al. Psychological Consequences of Rape on Women in 1991–1995 War in Croatia and Bosnia and Herzegovina. Croat Med J. 2006;47(1):67–75 PMID: 16489699. 16489699 PMC2080379

[25] Loncar M, Henigsberg N, Hrabac P. Mental health consequences in men exposed to sexual abuse during the war in Croatia and Bosnia. J Interpers Violence. 2010;25(2):191–203. 19641182 10.1177/0886260509334288

[26] Anderson K, et al. Predictors of Posttraumatic Growth among Conflict-Related Sexual Violence Survivors from Bosnia and Herzegovina. Conflict and Health. 2019;13:23 PMID: 31171935. 10.1186/s13031-019-0201-5 PMC6549258 31171935

[27] Salzman TA. Rape camps as a means of ethnic cleansing: religious, cultural, and ethical responses to rape victims in the former Yugoslavia. Hum Rights Q. 1998;20(2):348–78.

[28] OSCE. Combating Impunity for Conflict-Related Sexual Violence in Bosnia and Herzegovina: Progress and Challenges: An Analysis of Criminal Proceedings before the Court of Bosnia and Herzegovina between 2005 and 2013. Sarajevo: OSCE Mission to Bosnia and Herzegovina, 2014.

[29] Amnesty International. ’We Need Support Not Pity’. Last Chance for Justice for Bosnia’s Wartime Rape Survivors. Research report EUR 63/6679/2017. London: Amnesty International, 2017.

[30] Elisabeth D. Heineman, ed. Sexual Violence in Conflict Zones. Pennsylvania Studies in Human Rights. Philadelphia, Pa.; Oxford: University of Pennsylvania Press, 2011. 342.

[31] Ragnhild Nordås and Dara Kay Cohen. Conflict-Related Sexual Violence. Annu Rev Polit Sci. 2021;24:193–211.

[32] Berry Marie E. War, Women, and Power: From Violence to Mobilization in Rwanda and Bosnia-Herzegovina. Cambridge: Cambridge University Press; 2018. 10.1017/9781108236003.

[33] Priebe S, et al. Experience of human rights violations and subsequent mental disorders – a study following the war in the Balkans. Soc Sci Med. 2010;71(12):2170–7. 10.1016/j.socscimed.2010.09.029. 21041008 10.1016/j.socscimed.2010.09.029

[34] Devakumar D, et al. Mental Health of Women and Children Experiencing Family Violence in Conflict Settings: A Mixed Methods Systematic Review. Conflict and Health. 2021;15(1):74. 34654456 10.1186/s13031-021-00410-4 PMC8518246

[35] European Commission. GHSL Data Package 2023. JRC133256. Luxembourg: Publications Office of the European Union, 2023. 10.2760/098587.

[36] Ipsos. Life in Transition Survey IV: Technical Report. Technical report. London: European Bank for Reconstruction and Development (EBRD), 2023.

[37] American Institutes for Research (AIR). National Survey of Citizens’ Perceptions in Bosnia and Herzegovina 2023: Final Report. Technical report. Sarajevo: United States Agency for International Development (USAID), Bosnia and Herzegovina, 2024.

[38] Elm EV, et al. The Strengthening the Reporting of Observational Studies in Epidemiology (STROBE) Statement: Guidelines for Reporting Observational Studies. The Lancet. 2007;370(9596):1453–7 PMID: 18064739. 10.1016/S0140-6736(07)61602-X 18064739

[39] Judith Droitcour Miller. A New Survey Technique for Studying Deviant Behavior. PhD thesis. United States – District of Columbia: The George Washington University, 1984. 207.

[40] Traunmüller R, Kijewski S, Freitag M. The Silent Victims of Sexual Violence during War: Evidence from a List Experiment in Sri Lanka. Journal of Conflict Resolution. 2019;63(9):2015–42.

[41] Chapkovski P, Schaub M. Solid Support or Secret Dissent? A List Experiment on Preference Falsification during the Russian War against Ukraine. Research & Politics. 2022; 9(2). 20531680221108328.

[42] González B, Traunmüller R. The Political Consequences of Wartime Sexual Violence: Evidence from a List Experiment. Journal of Peace Research. 2024;61(6):1035–50.

[43] Rust KF, Rao J. Variance Estimation for Complex Surveys Using Replication Techniques. Statistical Methods in Medical Research. 1996;5(3):283–310. 8931197 10.1177/096228029600500305

[44] Gray A, Haslett S, Kuzmicich G. Confidence intervals for proportions estimated from complex sample designs. J Off Stat. 2004;20(4):705.

[45] Franco C, et al. Comparative study of confidence intervals for proportions in complex sample surveys. J Surv Stat Methodol. 2019;7(3):334–64. 31428658 10.1093/jssam/smy019 PMC6690503

[46] Efron B, Tibshirani RJ. An Introduction to the Bootstrap. New York: Chapman and Hall/CRC. 1994. 456.

[47] Agency for Statistics of Bosnia and Herzegovina. Projected population by age and sex. Population figures for Bosnia and Herzegovina, restricted to ages 18 and older. Accessed November 2025. 2020. (visited on 11/04/2025).https://pdo.bhas.gov.ba/1-7-1/

[48] Agency for Statistics of Bosnia and Herzegovina. Census of Population, Households and Dwellings in Bosnia and Herzegovina, 2013: Final Results. Agency for Statistics of Bosnia and Herzegovina Sarajevo, 2016.

[49] Alexandra Stiglmayer, ed. Mass Rape: The War against Women in Bosnia-Herzegovina. Trans. by Marion Faber. Lincoln: University of Nebraska Press, 1994. 232.

[50] Skjelsbæk I. The Political Psychology of War Rape: Studies from Bosnia and Herzegovina. Routledge; 2012.

[51] Johnson K, et al. Association of sexual violence and human rights violations with physical and mental health in territories of the Eastern Democratic Republic of the Congo. JAMA. 2010;304(5):553–62. 20682935 10.1001/jama.2010.1086

[52] Kuwert P, et al. Long-term effects of conflict-related sexual violence compared with non-sexual war trauma in female World War II survivors: a matched pairs study. Arch Sex Behav. 2014;43(6):1059–64. 24604012 10.1007/s10508-014-0272-8

[53] Jan Ilhan Kizilhan. Impact of Sexual Violation of ISIS Terror against Yazidi Women after Five Years. JSM Sex Med. 2020;4(1):1025.

[54] Svallfors S. Hidden casualties: the links between armed conflict and intimate partner violence in Colombia. Polit Gender. 2023;19(1):133–65.

[55] Boggiano B. Long-term effects of the Paraguayan War (1864-1870) on intimate partner violence. J Econ Behav Organ. 2024;222:177–224.

[56] Longombe AO, Claude KM, Ruminjo J. Fistula and Traumatic Genital Injury from Sexual Violence in a Conflict Setting in Eastern Congo: Case Studies. Reproductive Health Matters. 2008;16(31):132–41 PMID: 18513615. 18513615 10.1016/S0968-8080(08)31350-0

[57] Sarah K. Chynoweth et al. Characteristics and Impacts of Sexual Violence Against Men and Boys in Conflict and Displacement: A Multicountry Exploratory Study. Journal of Interpersonal Violence 2022;37:9–10 NP7470–NP7501. 10.1177/0886260520967132 33118459

[58] Woollett N et al. Understanding How Violence Is Transmitted across Generations: An in-Depth Multiple Case Study of Families in South Africa. Psychology of Violence 2025.

[59] Pham PN, Weinstein HM, Longman T. Trauma and PTSD Symptoms in Rwanda: Implications for Attitudes toward Justice and Reconciliation. JAMA: the journal of the American Medical Association. 2004;292(5):602–12 PMID: 15292086. 15292086 10.1001/jama.292.5.602

[60] Neugebauer R, et al. Post-Traumatic Stress Reactions among Rwandan Children and Adolescents in the Early Aftermath of Genocide. International Journal of Epidemiology. 2009;38(4):1033–45 PMID: 19204009. 19204009 10.1093/ije/dyn375

[61] Skjelsbæk I. Victim and survivor: narrated social identities of women who experienced rape during the war in Bosnia-Herzegovina. Fem Psychol. 2006;16(4):373–403.

[62] Priebe S, et al. Experience of Human Rights Violations and Subsequent Mental Disorders – A Study Following the War in the Balkans. Social Science & Medicine. 2010;71(12):2170–7. 21041008 10.1016/j.socscimed.2010.09.029

[63] Page D, Whitt S. Confronting Wartime Sexual Violence: Public Support for Survivors in Bosnia. Journal of Conflict Resolution. 2020;64(4):674–702.

[64] Stachow E. Conflict-Related Sexual Violence: A Review. BMJ Mil Health. 2020;166(3):183–7 PMID: 32447303. 32447303 10.1136/jramc-2019-001376

[65] Ba I, Bhopal RS. Physical, Mental and Social Consequences in Civilians Who Have Experienced War-Related Sexual Violence: A Systematic Review (1981–2014). Public Health. 2017;142:121–35. 27622295 10.1016/j.puhe.2016.07.019

[66] Rubini E, et al. Negative Consequences of Conflict-Related Sexual Violence on Survivors: A Systematic Review of Qualitative Evidence. International Journal for Equity in Health. 2023;22(1):227. 10.1186/s12939-023-02038-7 PMC10612192 37891663

[67] Spangaro J, et al. Mechanisms Underpinning Interventions to Reduce Sexual Violence in Armed Conflict: A Realist-Informed Systematic Review. Conflict and Health. 2015;9(1):19. 10.1186/s13031-015-0047-4 PMC4499895 26170898

[68] Marie E. Berry, Julianne Funk, and Nancy Good, eds. Healing and Peacebuilding after War Transforming Trauma in Bosnia and Herzegovina. Routledge Studies in Peace and Conflict Resolution. London: Taylor & Francis Ltd, 2023:226.

