# Supplementary material for: Exposure to wartime sexual violence in Bosnia and Herzegovina: nationally representative prevalence 30 years after the 1992–1995 war
Source: Confl Health. 2025 Dec 23;19:96. doi: 10.1186/s13031-025-00741-6 (PMC12754861; doi:10.1186/s13031-025-00741-6)
Supplement: Supplementary file 1 [file 13031_2025_741_MOESM1_ESM.pdf]

Supplementary Information (SI) for "Exposure to wartime sexual violence in Bosnia and Herzegovina: Nationally representative prevalence 30 years after the 1992–1995 war"

Table of Contents

A Declarations **1**

B Additional information on survey **2**

Construction of strata . . . 2

Survey implementation . . . 3

C Full summary statistics **4**

D Psychosocial correlates of personal and community WTSV **5**

E Question wording **6**

Survey question wording . . . 6

F STROBE reporting checklist **8**

A Declarations

Ethics approval and consent to participate

This study was approved by the WZB Berlin Social Science Center Research Ethics Review Board (Decision Nr. 2024/03/240). All participants received an explanation of the study procedures and provided informed consent before taking part in the interview. Interviews were conducted by trained enumerators in Bosnian using a pre-tested questionnaire. Given the sensitive nature of the survey, several measures were taken to ensure respondent safety and privacy. Respondents were informed that participation was voluntary and that they could skip any question. Sensitive items were self-completed on the tablet to maximize privacy, and the survey software prevented enumerators from returning to these items after confirmation. Enumerators received dedicated training on sensitive interviewing and recognizing emotional distress. A psychologist specializing in wartime trauma was on stand-by during data collection; no referrals were required during fieldwork.

Consent for publication

Not applicable.

Authors' contributions

Conceptualization: All authors; Methodology: MS, LK; Survey instrument development: All authors; Fieldwork supervision: All authors; Data collection: All authors; Formal analysis and interpretation: AGF, LK, MS; Writing – original draft: MS; Writing – review & editing: All authors; Guarantor: MS.

Competing interests

The authors declare no competing interests. MS (University of Hamburg) received support from the German Research Foundation (DFG). The funder had no role in the design, implementation, analysis, or publication of this study.

Funding

This study was supported by the German Research Foundation (DFG), Grant No. SCHA 2170/2-1.

Data availability

De-identified survey data and replication code are available on OSF at https://osf.io/7zksg/overview?view_only=54ffb03505ca4d4c8674dea25b46a75e. Sensitive identifiers have been removed to protect respondent confidentiality. The data will be made fully public upon publication of this article.

Acknowledgments

We thank the respondents for sharing their experiences, the staff of MIB Kantar Sarajevo for the meticulous survey implementation, and the interviewers for their work. Our two anonymous reviewers and Shari Kupilas provided very helpful comments. This article emerged from discussions with civil society organizations working on sexual violence during a workshop held in Sarajevo in 2025, where the lack of reliable prevalence data was identified as a critical gap.

B Additional information on survey

Recruitment and participation

In total, 5,288 individuals were approached as part of the household recruitment process. Of these, 3,073 contact attempts were unsuccessful because no eligible respondent was available or contact could not be established, and 156 interviews were excluded for quality reasons. The resulting analytical sample comprises 2,059 completed interviews. This corresponds to a contact rate of 40.0%, a cooperation rate of 92.9% among contacted individuals, and an overall response rate of 38.9%. Table S1 provides the full breakdown of contact outcomes.

In addition to the main sample, we interviewed around 600 family members of our original respondents as part of our wider data collection effort. Since this recruitment did not follow random recruitment, these interviews are not used in the present analysis.

Table S1: Survey contact and cooperation rates)

	**Original respondents**
Total contact attempts	5,288
Unsuccessful or excluded	
Unsuccessful recruitment	3,073
Quality exclusions (interviewer)	83
Quality exclusions (interview)	65
Quality exclusions (technical)	8
Successfully contacted (eligible & reached)	2,215
Completed interviews	2,059
**Contact rate** (contacted / attempts)	40.0%
**Cooperation rate** (completed / contacted)	92.9%
**Response rate** (completed / attempts)	38.9%

Construction of strata

To ensure that areas most likely to have experienced wartime sexual violence (WTSV) were sufficiently represented in the sample, we constructed a settlement-level risk index and oversampled from high-risk settlements. This strategy was motivated by extensive qualitative and documentary evidence

showing that much of the sexual violence during the Bosnian war occurred in and around detention locations where civilians were held and abused [1–5].

We operationalized settlement-level risk using data on wartime detention locations compiled by the Association for Association for Transitional Justice, Accountability and Remembrance (TPOS). For each settlement i, we identified the three closest detention sites in which civilians belonging to the settlement's majority ethnic group had been detained and victimized. Distances between settlement centroids and detention sites were computed using road-network travel distances derived from OpenStreetMap.

The risk index R for settlement i was defined as:

$$R_i = \sum_{j=1}^{j=3} \log_{10} \mathrm{Detainees}(j) - \sum_{j=1}^{j=3} \log_{10} \mathrm{Distance}(i, j)$$

where j indexes the three nearest relevant detention locations. Risk thus increases when nearby sites held larger numbers of detainees and when those sites were geographically closer to the settlement. After computing the index for all settlements, we classified settlements scoring at or above the 75th percentile as "high-risk," with the remainder forming the "low-risk" stratum. The sampling frame was then constructed by drawing an equal number of settlements from each stratum. All analyses apply design weights that undo this oversampling to recover population-representative prevalence estimates.

Survey implementation

Survey implementation proceeded as planned. No changes were made to the study design or eligibility criteria after commencement. Pre-specified outcomes followed the pre-analysis plan, and no modifications were introduced during data collection. Because the study was a household survey with a fixed target sample, no interim analyses or stopping guidelines were planned or implemented.

C Full summary statistics

Table S2: Summary statistics for sample demographics, victimization, and well-being outcomes

	Mean	SD	Min	Max	Count
**Sample demographics**					
Female	0.49	0.50	0.00	1.00	2,059
Age	45.91	16.49	18.00	85.00	2,059
Bosniak	0.50	0.50	0.00	1.00	2,059
Serb	0.35	0.48	0.00	1.00	2,059
Croat	0.15	0.36	0.00	1.00	2,059
Other	0.01	0.07	0.00	1.00	2,059
**Wartime sexual violence (WTSV)**					
WTSV (self)	0.02	0.13	0.00	1.00	968
WTSV (family)	0.06	0.24	0.00	1.00	2,059
WTSV (community)	0.11	0.32	0.00	1.00	2,059
**Other victimization events**					
House, business, or fields destroyed (self)	0.16	0.37	0.00	1.00	968
House, business, or fields destroyed (family)	0.26	0.44	0.00	1.00	2,059
House, business, or fields destroyed (community)	0.39	0.49	0.00	1.00	2,059
Forced out of home (self)	0.21	0.40	0.00	1.00	968
Forced out of home (family)	0.28	0.45	0.00	1.00	2,059
Forced out of home (community)	0.38	0.48	0.00	1.00	2,059
Tortured (self)	0.02	0.15	0.00	1.00	968
Tortured (family)	0.09	0.28	0.00	1.00	2,059
Tortured (community)	0.17	0.38	0.00	1.00	2,059
Detained (self)	0.02	0.14	0.00	1.00	968
Detained (family)	0.07	0.26	0.00	1.00	2,059
Detained (community)	0.17	0.37	0.00	1.00	2,059
Shot, shelled, bombed (self)	0.17	0.38	0.00	1.00	968
Shot, shelled, bombed (family)	0.23	0.42	0.00	1.00	2,059
Shot, shelled, bombed (community)	0.31	0.46	0.00	1.00	2,059
Seriously wounded (self)	0.04	0.20	0.00	1.00	968
Seriously wounded (family)	0.22	0.41	0.00	1.00	2,059
Seriously wounded (community)	0.47	0.50	0.00	1.00	2,059
Killed / killed in combat (self)	0.00	0.00	0.00	0.00	968
Killed / killed in combat (family)	0.19	0.39	0.00	1.00	2,059
Killed / killed in combat (community)	0.45	0.50	0.00	1.00	2,059
Disappeared (self)	0.00	0.00	0.00	0.00	968
Disappeared (family)	0.08	0.28	0.00	1.00	2,059
Disappeared (community)	0.18	0.38	0.00	1.00	2,059
Sentenced to death / executed (self)	0.00	0.00	0.00	0.00	968
Sentenced to death / executed (family)	0.07	0.25	0.00	1.00	2,059
Sentenced to death / executed (community)	0.12	0.33	0.00	1.00	2,059
**Well-being and psychosocial outcomes**					
Happiness	3.68	0.78	1.00	5.00	2,059
General health	3.40	1.07	1.00	5.00	2,059
Sleep quality	4.00	1.02	1.00	5.00	2,053
Emotional health	4.09	0.96	1.00	5.00	2,042
Domestic violence	0.09	0.28	0.00	1.00	2,059

Note: Weighted summary statistics for core demographic, victimization, and psychosocial variables. “Self” refers to the respondent, “family” to immediate family members, and “community” to events affecting others in the respondent’s community.

D Psychosocial correlates of personal and community WTSV

Figure S1: Correlation of *personal* exposure to wartime sexual violence and measures of well-being and domestic violence

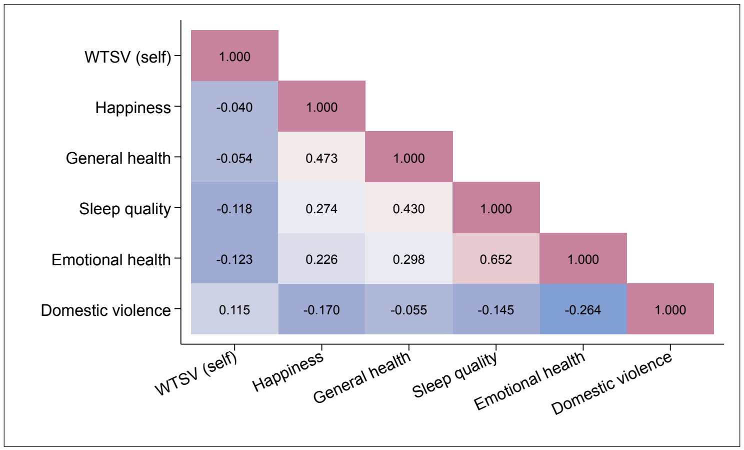

Note: Pairwise correlations between personal exposure to wartime sexual violence (WTSV) and indicators of well-being and domestic violence. All correlations statistically significant at $p < 0.01$.

Figure S2: Correlation of *community* exposure to wartime sexual violence and measures of well-being and domestic violence

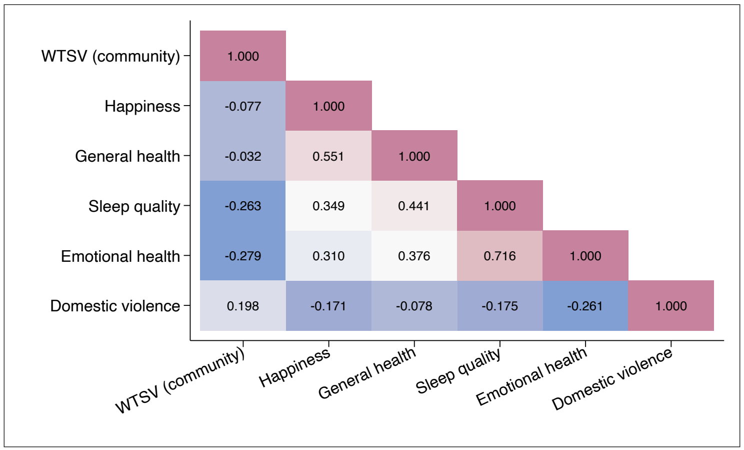

Note: Pairwise correlations between community exposure to wartime sexual violence (WTSV) and indicators of well-being and domestic violence. All correlations statistically significant at $p < 0.01$.

E Question wording

Survey question wording

Table S3: Question wording for key variables

Variable Name	Question Text	Answer Options
**Demographics**		
Sex (Female)	What is your sex?	1 Male; 2 Female; 3 Other/Diverse; −88 Refused. *Note:* Recoded as a binary indicator where 1 = Female and 0 = Male; "Other" and "Refused" not chosen by any respondent.
Ethnicity	What is your ethnicity?	1 Bosniak; 2 Serb; 3 Croat; 4 Other; 5 Prefer not to say; 6 Don't think in those terms; −88 Refused. *Note:* Recoded into four categories: 1 = Bosniak; 2 = Serb; 3 = Croat; 4 = Other/refused/not. When "Prefer not to say" or "Don't think in those terms" was selected, responses were replaced by ascribed ethnicity (below) when available.
Ascribed ethnicity	How would other people categorize your ethnicity? *(Asked if 5 or 6 in question above.)*	1 Bosniak; 2 Serb; 3 Croat; 4 Other; −88 Refused. *Note:* Used to impute ethnicity for respondents selecting "Prefer not to say" or "Don't think in those terms."
**Victimization (older respondents, $q1\_age > 43$)**		
Events during the war (self, family, community)	During the period of the war in the 1990s, which of the following, if any, happened to you, members of your family, or your community? (Respondent completes privately.)	(a) Had house, business, or fields destroyed; (b) Forced out of home; (c) Tortured; (d) Taken to detention/concentration camp; (e) Subjected to sexual violence; (f) Shot, shelled, or bombed; (g) Seriously wounded; (h) Killed/killed in combat; (i) Disappeared; (j) Sentenced to death/executed. *Note:* Refused coded as missing.
Answer options for each item		1 Me; 2 Someone in my family; 3 Someone in my community; −88 Refused.
**Victimization (younger respondents, $q1\_age <= 43$)**		
Events during the war (family, community)	During the war in the 1990s, which of the following, if any, happened to members of your family or community? (Respondent completes privately.)	(a) Had house, business, or fields destroyed; (b) Forced out of home; (c) Tortured; (d) Taken to detention/concentration camp; (e) Subjected to sexual violence; (f) Shot, shelled, bombed; (g) Seriously wounded; (h) Killed/killed in combat; (i) Disappeared; (j) Sentenced to death/executed. *Note:* Refused coded as missing.
Answer options for each item		1 Happened to someone in my family; 2 Happened to someone in my community; −88 Refused. *Note:* Refused coded as missing.

Continued on next page

Continued from previous page

Variable Name	Question Text	Answer Options
**List experiment on WTSV exposure**		
Self treat list (respondents, *age* > 43 only)	During the war in the 1990s, how many of the following happened to *you*? You don't have to tell which, just how many (0–4):	I had to leave my home; I was part of a prisoner's exchange; I witnessed someone being killed; I was subjected to sexual violence. *Note:* Responses range from 0 to 4.
Family treat list	During the war in the 1990s, how many of the following happened to *members of your family*? (0–4)	Someone in my family had to leave their home; was part of a prisoner's exchange; witnessed someone being killed; was subjected to sexual violence. *Note:* Responses range from 0 to 4.
Self control list (no SV, respondents, *age* > 43 only)	During the war in the 1990s, how many of the following happened to you? (0–3)	I had to leave my home; I was part of a prisoner's exchange; I witnessed someone being killed. *Note:* Responses range from 0 to 3.
Family control list (no SV)	During the war in the 1990s, how many of the following happened to family members? (0–3)	Someone in my family had to leave their home; was part of a prisoner's exchange; witnessed someone being killed. *Note:* Responses range from 0 to 3.
**Outcomes and well-being**		
Happiness	All things considered, how happy or unhappy would you say you are with your life at the moment?	1 Very unhappy; 2 Fairly unhappy; 3 Neither happy nor unhappy; 4 Fairly happy; 5 Very happy; −88 Refused. *Note:* Refused coded as missing.
General health	In general, would you say your health is...	1 Poor; 2 Fair; 3 Good; 4 Very good; 5 Excellent; −88 Refused. *Note:* Refused coded as missing.
Sleep quality	Difficulty falling or staying asleep, nightmares	1 Never; 2 Rarely; 3 Occasionally; 4 Frequently; 5 Very frequently; 6 Don't remember/know; −88 Refused. *Note:* Scale reversed so that higher values indicate better sleep quality. "Don't remember/know" and Refused coded as missing.
Emotional health	Sudden mood changes, feeling a lack of control over own emotions or thoughts	1 Never; 2 Rarely; 3 Occasionally; 4 Frequently; 5 Very frequently; 6 Don't remember/know; −88 Refused. *Note:* Scale reversed so that higher values indicate better emotional health. "Don't remember/know" and Refused coded as missing.
Domestic violence	Have you ever experienced or observed violence in your family context? (physical or emotional abuse)	1 Yes, experienced as a child; 2 Yes, experienced in my own relationship(s); 3 Yes, observed among my parents; 4 Yes, observed among my grandparents; 5 No, none of the above; −88 Refused. *Note:* Binary indicator created as 1 if respondent reported experiencing or observing violence as a child or in own relationship (any of items 1–2), and 0 otherwise. Refused coded as missing.

F STROBE reporting checklist

Table S4: STROBE 2007 reporting checklist for observational (cross-sectional) studies

Section/Topic	Item Nr.	Checklist Item	Page Nr./Note
**Title and abstract**	1a	Indicate the study design with a commonly used term in the title or abstract	Title; 1
	1b	Provide an informative, balanced summary of what was done and found	Abstract, p. 1
**Introduction**	2	Explain the scientific background and rationale for the investigation being reported	2
	3	State specific objectives, including any prespecified hypotheses	2
**Methods**	4	Present key elements of study design early in the paper	2
	5	Describe the setting, locations, and relevant dates, including periods of recruitment and data collection	2
	6a	Give the eligibility criteria and methods of selection of participants	2
	6b	For matched studies, give matching criteria and number of controls per case (n/a here)	–
	7	Clearly define all outcomes, exposures, predictors, potential confounders, and effect modifiers	3
	8	For each variable of interest, give data sources and details of assessment (measurement); describe comparability across groups	3, 7
	9	Describe any efforts to address potential sources of bias	3, 7
	10	Explain how the study size was arrived at	2
	11	Explain how quantitative variables were handled in the analyses and which groupings were chosen and why	4
	12a	Describe all statistical methods, including those used to control for confounding	4
	12b	Describe any methods used to examine subgroups and interactions	5
	12c	Explain how missing data were addressed	2
	12d	If applicable, explain how matching of cases and controls was addressed (n/a)	–
	12e	Describe any sensitivity analyses performed	7, 7
**Results**	13a	Report numbers of individuals at each stage—eligible, included, completing interview, analyzed	2
	13b	Give reasons for non-participation at each stage	2
	13c	Consider use of a flow diagram (not used)	–
	14a	Give characteristics of study participants (demographic, clinical, social) and information on exposures and potential confounders	5
	14b	Indicate number of participants with missing data for each variable of interest	2
	15	Report numbers in each exposure category, or summary measures of exposure	5, 5
	16a	Give unadjusted and, if applicable, adjusted estimates and their precision (e.g., 95% CI), making clear which confounders were adjusted for	5, 7, 7
	16b	Report category boundaries when continuous variables were categorized	5
	16c	If relevant, translate estimates into absolute risks for a meaningful period (n/a)	–
	17	Report other analyses—e.g., subgroup and sensitivity analyses	5, 7, 7
**Discussion**	18	Summarize key results with reference to study objectives	8

Continued on next page

Continued from previous page

**Section/Topic**	**Item Nr.**	**Checklist Item**	**Page Nr.**
	19	Discuss limitations of the study, taking into account sources of bias or imprecision, and discuss their direction and magnitude	8
	20	Give a cautious overall interpretation considering objectives, limitations, and results from similar studies	8
	21	Discuss the generalizability (external validity) of the study results	8
**Other information**	22	Give the source of funding and the role of the funders for the present study	1
